# Molecular signatures of alternative reproductive strategies in a facultatively social hover wasp

**DOI:** 10.1111/mec.17217

**Published:** 2023-11-28

**Authors:** Benjamin A. Taylor, Daisy Taylor, Alexandrina Bodrug‐Schepers, Francisco Câmara Ferreira, Nancy Stralis‐Pavese, Heinz Himmelbauer, Roderic Guigó, Max Reuter, Seirian Sumner

**Affiliations:** ^1^ Centre for Biodiversity & Environment Research University College London London UK; ^2^ Department of Genetics, Evolution & Environment University College London London UK; ^3^ School of Biological Sciences University of Bristol Bristol UK; ^4^ Department of Biotechnology University of Natural Resources and Life Sciences Vienna Austria; ^5^ Centre for Genomic Regulation Barcelona Institute of Science and Technology Barcelona Spain; ^6^ Universitat Pompeu Fabra Barcelona Spain; ^7^ Centre for Life's Origins and Evolution University College London London UK

**Keywords:** behavior/social evolution, eusociality, social insects, sociogenomics, transcriptomics

## Abstract

Social insect reproductives and non‐reproductives represent ideal models with which to understand the expression and regulation of alternative phenotypes. Most research in this area has focused on the developmental regulation of reproductive phenotypes in obligately social taxa such as honey bees, while relatively few studies have addressed the molecular correlates of reproductive differentiation in species in which the division of reproductive labour is established only in plastic dominance hierarchies. To address this knowledge gap, we generate the first genome for any stenogastrine wasp and analyse brain transcriptomic data for non‐reproductives and reproductives of the facultatively social species *Liostenogaster flavolineata*, a representative of one of the simplest forms of social living. By experimentally manipulating the reproductive ‘queues’ exhibited by social colonies of this species, we show that reproductive division of labour in this species is associated with transcriptomic signatures that are more subtle and variable than those observed in social taxa in which colony living has become obligate; that variation in gene expression among non‐reproductives reflects their investment into foraging effort more than their social rank; and that genes associated with reproductive division of labour overlap to some extent with those underlying division of labour in the separate polistine origin of wasp sociality but only explain a small portion of overall variation in this trait. These results indicate that broad patterns of within‐colony transcriptomic differentiation in this species are similar to those in Polistinae but offer little support for the existence of a strongly conserved ‘toolkit’ for sociality.

## 1 INTRODUCTION

Understanding how and why indirect fitness strategies have evolved and are regulated has long been a major question in evolutionary biology. In most social taxa, the evolution of indirect modes of reproduction can ultimately be explained in terms of inclusive fitness theory: altruistic behaviour evolves because altruists increase the fitness of individuals to whom they are related (Foster et al., 2006; Hamilton, 1963). The question of how non‐reproductive and reproductive phenotypes can arise from a shared genome at a proximate level is, however, less well‐resolved. Research over the last two decades has begun to reveal how alternative reproductive strategies are produced through differential expression of shared molecular machinery (e.g. Morandin et al., 2019; Patalano et al., 2015; Qiu et al., 2018, 2022), but most research in this area has focused on social insects that always live in colonies comprised of mutually dependent individuals, each of whom is committed to a specific and morphologically distinct queen or worker phenotype for life. The molecular basis of developmental caste differentiation in such species has been the focus of numerous studies, which have revealed that queens and workers exhibit strongly and consistently divergent patterns of gene expression (e.g. He et al., 2019; Morandin et al., 2015; Qiu et al., 2018, 2022; Warner et al., 2019).

Despite the ecological importance of taxa with permanent morphologically differentiated castes (Wilson, 1990), these taxa are relatively taxonomically limited. In insects, for example, such permanent differentiation is found only in the ants, honey bees, stingless bees, bumble bees, vespine wasps and higher termites (Boomsma & Gawne, 2018). Simpler forms of social organization, in which individuals retain the ability to switch between reproductive and non‐reproductive roles within their lifetime, are much more common in both insects (e.g. polistine and stenograstrine wasps; halictid and xylocopid bees) and cooperatively breeding vertebrates (e.g. meerkats and long‐tailed tits). Importantly, non‐reproductive individuals in these lineages with open societies and flexible dominance hierarchies retain the ability to mate and to adjust their reproductive physiology, and are potential successors to the dominant reproductive if she dies or if an alternative nesting opportunity arises (Field et al., 2006; Leadbeater et al., 2011; Smith et al., 2009). Such individuals can be described as ‘hopeful reproductives’ that invest in altruistic behaviour while waiting for opportunities to reproduce (West‐Eberhard, 1975).

There are also social lineages where sociality itself has remained facultative because solitary colonies coexist in the same breeding population with social colonies that maintain a hierarchy of actual and hopeful reproductives. The flexible and reversible nature of their reproductive strategies makes these facultatively social insects ideal models for understanding the emergence of the simplest forms of reproductive division of labour (Kronauer & Libbrecht, 2018; Shell & Rehan, 2018). For example, facultatively social bee taxa have been used to provide evidence for ‘molecular ground plan’ hypotheses (Kapheim et al., 2012, 2020) and to test the long‐standing sociogenomic prediction that reproductive‐biased genes should be relatively ancient and conserved compared to worker‐biased genes (Jones et al., 2017). Genomic studies using facultatively social insects outside of the Anthophila (bees) are lacking, however. Accordingly, we have a limited and potentially biased understanding of the proximate nature of alternative reproductive strategies in facultatively social insects, that is, how the highly flexible alternative fitness strategies in these species are produced from a shared genome and how such plasticity is regulated (Taylor et al., 2021). Specifically, we would like to know whether the degree of molecular differentiation present among the uncommitted reproductive roles of facultatively social species is similar to that which exists in species where roles are expressed more stably. In addition, few studies have addressed the question of the degree to which continuous variation in individual‐level investment in reproductive and non‐reproductive helping activities is reflected at the molecular level. Species with reproductive queues that predict investment into altruistic behaviours should be particularly suitable for the study of this topic.

Here, we address these questions by generating the first genome for a member of the Stenogastrinae, a taxon that represents an independent evolutionary origin of facultative eusociality (Bank et al., 2017) separate from all other social wasps, and then conducting the first transcriptomic analyses for *Liostenogaster flavolineata*, the best‐studied member of this clade. As in other facultatively social species, many *L. flavolineata* individuals spend part of their lives as non‐reproductive helpers, but such individuals may transition at some point to a reproductive role, shifting between indirect and direct fitness strategies (Bridge & Field, 2007; Field et al., 2006).

*Liostenogaster flavolineata* colonies are small (2–10 individuals; Bridge & Field, 2007) and consist of strictly age‐determined dominance hierarchies, with the oldest non‐reproductive individual in a colony being first in line to mate and replace the reproductive if she dies. Helper females are unmated, but males are produced throughout the year and disperse soon after eclosion to look for virgin females on established nests (Field, 2008). It is therefore likely that females can mate and begin producing diploid offspring soon after being promoted to a dominant role, without needing to leave the nest to seek a mate. Subordinate females within a colony are close kin, with a mean relatedness of 0.5 (Field et al., 2006; Sumner et al., 2002). Because they are more likely to attain a reproductive role in the near future, older (higher ranked) individuals exhibit a reduced foraging effort relative to younger (lower ranked) individuals, indicating a shift away from investment into risky behaviours as the chances of future reproductive opportunities improve (Bridge & Field, 2007; Field et al., 2006). It is also possible that subordinate females may leave to found their own nests. At any given time, however, only a single individual within a colony acts as an egg layer, so *L. flavolineata* also exhibits the strong reproductive division of labour that is the defining characteristic of insect sociality (Sumner et al., 2002).

Based on these biological features, *L. flavolineata* represents an ideal model with which to address the outstanding questions outlined above and to shed light on the molecular correlates of adult phenotypic plasticity for breeder and reproductive roles. By sequencing the genome of this species, the first for any member of the Stenogastrinae, conducting controlled experiments to manipulate social hierarchy and fitness‐related behaviour, and examining in detail the influence of social rank and altruistic activity upon brain transcription, we generate novel insights into the molecular origins of plastic social phenotypes. Our data will also provide the basis for future investigations into the proximate basis of sociality in this independent social lineage.

## 2 METHODS

### 2.1 Field monitoring and behavioural experiments

#### 2.1.1 Experimental set‐up

Fieldwork was undertaken in Fraser's Hill, Malaysia between January and April 2017. *L. flavolineata* nests from aggregations situated in under‐road culverts were selected for study on the basis of the presence of multiple pupal caps and observation of active egg laying. Over 2 days, all individuals on each nest were given a unique combination of coloured paint marks to facilitate subsequent individual‐level identification. Confirmation that all wasps had been successfully marked was achieved by censuses of colony members at night, when all individuals are present in the nest. Brood was mapped in each nest to confirm the presence of an egg layer and to identify pupae that would shortly hatch. Reproductives were identified by observation of egg laying or through censuses to determine foraging effort: reproductives rarely leave the nest in this species (Cant & Field, 2001; Field & Foster, 1999; Shreeves & Field, 2002). Nests were monitored every day to measure foraging effort and identify newly emerging wasps. Newly emerged colony members were identified by the co‐appearance of an unmarked wasp and a hatched pupal cell. Once identified, newly emerged individuals were left on the nest for 3 days before marking, to avoid interfering with their nest orientation flights.

Focal nests (*n* = 28) consisting of 3–5 wasps of known age and rank were generated by removing unmarked wasps and/or wasps of unknown age; such colony sizes are typical of natural nests in this species (Field et al., 2000; Shreeves & Field, 2002). To achieve this, nests were censused daily until 2–4 new individuals had emerged. These newly emerged individuals, together with the nest's reproductive (Rank 1), were used as the focal individuals for the rest of the study. To generate nests of comparable colony sizes and with non‐reproductives of known ages, all other wasps were removed at dawn on the day following the emergence of the requisite number of focal wasps. For 10 days following this manipulation, nests were censused every ~30 min during peak foraging hours (07:00–11:00) to quantify time spent off the nest for each individual. Time spent off the nest is thought to be a reliable proxy for foraging effort in *L. flavolineata*: older individuals are more likely to inherit the position of egg layer and are therefore expected to invest less in risky foraging behaviour (Bridge & Field, 2007; Field et al., 2006). From this initial manipulation until the end of the experiment, additional wasps that emerged from the nest were removed to ensure that colony sizes remained constant. Thus, at the end of the 10‐day period, the rank and average time spent off‐nest were known for each individual from 28 focal nests consisting of a single reproductive (exact age unknown but >2 months old) and 2–4 non‐reproductives of known ages (within‐rank age range ≈ 5 days). These preliminary manipulations allowed us to generate a starting population of nests for which factors such as age, colony size, brood number and foraging effort were quantified and standardized for use in our experiment and analyses.

#### 2.1.2 Experiments and sampling

Using an experimental design based on that employed by Field et al. (2006), we performed manipulations in which the second‐ranked (Rank 2) or third‐ranked (Rank 3) individual from each nest was removed in order to promote lower‐ranked wasps to a higher rank within the colony hierarchy. To promote non‐reproductive wasps on each nest, either from Rank 3 to Rank 2 or from Rank 4 to Rank 3, at dawn on day 11, a single focal wasp was removed from each of the 28 nests and placed directly into RNAlater (Thermo Fisher Scientific). The removed individual was always of either Rank 2 (*n* = 15; age mean ± SD = 30.3 ± 3.1 days) or Rank 3 (*n* = 7; age mean ± SD = 25.3 ± 1.4 days). To ensure that the ratio of helpers to brood remained constant despite the loss of a helper, brood was also removed from the nests at this time. Brood was divided into three categories: eggs, small/medium larvae and large larvae. A proportion, *R*/*N* (where *R* is the number of adults removed and *N* is the original number of wasps), of each category was removed using fine tweezers. Pupae, which do not require feeding, were not removed. Nests were given 48 h to settle, after which censuses were performed daily for 5 days using the same methodology as previously. At the end of the 5‐day censusing period, all wasps were removed from the nest before dawn: individuals' heads were placed directly into RNA later for gene expression analysis, and their bodies into 95% EtOH for dissection. The ages of non‐reproductives of the same final rank collected at this stage that were subsequently sequenced were approximately age‐matched across nests (age mean ± SD: Rank 2 = 33.3 ± 4.3 days; Rank 3 = 27.0 ± 1.6 days; Rank 4 = 22.8 ± 1.5 days). The ovarioles of each individual were dissected and the number of developed eggs counted. The mating status of each individual was assessed by examining the spermatheca for the presence of sperm.

This design allowed us to compare gene expression between reproductives (Rank 1) and non‐reproductives (Ranks 2–5). The design also enabled us to assess the extent to which variation in gene expression varied among non‐reproductives of different ranks (Ranks 2–5) and, therefore, with investment into risky foraging behaviour. If rank and/or foraging effort are reflected at the level of brain transcription, then we expected that fine‐scale molecular differentiation among non‐reproductive ranks (i.e. Ranks 2–5) might be as profound as that between reproductives and non‐reproductives (i.e. Rank 1 vs. Ranks 2–5). Finally, our chosen experimental design allowed us to compare the brain gene expression patterns of individuals that had been promoted in rank to those which had not undergone promotion. We predicted that individuals that had been promoted from Rank 3 to Rank 2 would exhibit a concomitant shift in rank‐ and foraging‐related gene expression: if this were the case, then individuals promoted from Rank 3 to Rank 2 should more closely match the expression patterns of unmanipulated Rank 2 individuals than of unmanipulated Rank 3 individuals.

### 2.2 Genomic and transcriptomic analyses

#### 2.2.1 Genome sequencing and assembly

Complete methods and results for the assembly are given in Appendix S1. In brief, DNA was extracted from a single haploid *L. flavolineata* male using the DNeasy Blood & Tissue Kit (Qiagen) according to the manufacturer's instructions. DNA quantification was performed with a Qubit 3.0 fluorometer using the dsDNA BR assay kit (Thermo Fisher), and DNA integrity was monitored on an agarose gel. A paired‐end (PE) sequencing library with a peak insert size of 535 bp was constructed using 200 ng of genomic DNA with a TruSeq Nano LT library preparation kit (Cat # FC‐121‐4002, Illumina) according to the kit supplier's instructions. A mate‐pair (MP) library with a peak span size of 1450 bp and a mean span size of 1027 bp was prepared from 570 ng of genomic DNA by tagmentation using an MP library preparation kit (Cat # FC‐132‐1001, Illumina), without size selection. The MP library was amplified using 12 cycles of PCR. The quality and quantity of the libraries were checked on a DNA 1000 chip on the Agilent Bioanalyzer 2100 (Agilent).

Illumina sequencing was performed on a HiSeq 2500 instrument utilizing v4 Illumina sequencing chemistry, combined with a 2 × 125 cycle sequencing recipe. Raw sequencing data underwent quality control with FastQC (Andrews, 2010). Thereafter, trimmomatic (Bolger et al., 2014) was employed for data filtering based on phred scores, using the following parameters: LEADING:25 TRAILING:25 SLIDINGWINDOW:10:25 MINLEN:36. Genome assemblies were performed using SOAPdenovo_v2.04 (Luo et al., 2012). Pre‐assemblies were first calculated based on PE reads which were assembled either as single reads or as pairs (Dohm et al., 2014), in order to assess the insert size distribution (PE reads) and span size distribution (MP reads) of the sequencing libraries, respectively. Bowtie2 (Langmead & Salzberg, 2012) was used with an insert size interval between 100 and 1200 and 1 million PE read pairs were sampled to estimate the library insert size. To estimate the span size of the MP read pairs, bowtie2 was used on an assembly version using the paired‐end library as pairs and an insert size interval between 100 and 20,000, and sampled 1 million MP read pairs. Using the determined library insert size and MP span size as parameters for the assembly run, several assemblies were calculated from the quality‐filtered sequencing reads by varying the k‐mer size parameter between 23 and 125. An assembly calculated with k‐mer size 69 was the best performing in terms of assembly metrics as assessed by QUAST (Gurevich et al., 2013).

BUSCOv3 (Simão et al., 2015; Waterhouse et al., 2018) was used in genome mode with the hymenoptera_odb9 lineage and honeybee1 species to assess assembly completeness (blast 2.2.30, AUGUSTUS 3.2.1). Metrics of the final assembly were determined with custom scripts, taking only sequences larger than 500 bp into account. Jellyfish 2.2.10 (Marçais & Kingsford, 2011) was used to determine genome size based on the quality‐filtered Illumina PE sequencing reads. Bioawk was used to retrieve the GC content of all the reads as well as non‐overlapping 125 nt segments of the final assembly lacking undetermined bases (no unknown nucleotide “N”).

#### 2.2.2 Gene expression quantification

Brain tissue was extracted from the heads of a subset of individual focal samples, all from manipulated nests, and RNA was extracted using an RNeasy Mini Kit (Qiagen) according to the manufacturer's instructions. Library preparation was performed by Novogene Co. followed by sequencing on an Illumina HiSeq 2000 platform with 150‐bp paired‐end reads to a depth of 30 million reads/sample. Transcript filtering, trimming, alignment and quantification were performed with the aid of Nextflow using the default options provided by the nf‐core/rnaseq pipeline v1.4.2 (Ewels et al., 2020): adapter and quality trimming were performed using TrimGalore (Krueger, 2015), followed by removal of ribosomal sequences using SortMeRNA (Kopylova et al., 2012); read alignment against the *L. flavolineata* genome was performed using STAR (Dobin et al., 2013) followed by quantification using Salmon (Patro et al., 2017); aligned reads were assembled into genes using StringTie2 (Kovaka et al., 2019). Quality control (QC) checks identified six samples (three Rank 1 s; two Rank 2 s; one Rank 4) as exhibiting low sequencing quality, and these samples were excluded from subsequent analyses as a result.

#### 2.2.3 Differential expression analysis

Differential expression differences between groups of individuals were modelled in R with the DESeq2 package (Love et al., 2014). Prior to differential expression analysis, read counts were subjected to a minimal round of filtering to remove any gene that was not expressed at a minimum of one count/sample in at least one group of samples (as grouped by original rank). Following this filtering, 11,258/14,095 (79.9%) genes remained. More stringent filtering was deemed unnecessary at this stage as DESeq2 applies its own independent filters to remove genes with no power (Love et al., 2014).

For most analyses, we applied univariate models (expression ~ trait) with either categorical (e.g. reproductive role) or continuous (e.g. age) independent variables. Because rank and foraging effort are expected to be strongly collinear in this system, we also sought to identify genes differentially expressed with respect to these variables independent of one another. To do so, we generated a linear model of rank on foraging effort and then ran an additive DESeq2 model with foraging effort plus the residuals of rank on foraging effort taken from the aforementioned linear model. We considered genes differentially expressed with the residuals of rank on foraging effort in this model to be associated with rank, independent of the effects of foraging effort. The same process (with rank and foraging effort swapped) was used to identify genes differentially expressed with foraging effort independent of rank.

Except where otherwise stated, differential expression was calculated relative to a baseline fold change of 0. Otherwise, we applied a log(2) fold change threshold of 1.5, equivalent to a ~58% change in expression between groups. This threshold was applied during testing rather than post‐hoc, that is, we tested differential expression using this fold change as the baseline, a more conservative approach than merely checking whether the log fold change exceeded the given threshold. Genes were considered differentially expressed between conditions if *p* < .05 after false discovery rate correction according to the Benjamini–Hochberg procedure.

### 2.3 Gene ontology (GO) enrichment analysis

To perform GO enrichment analysis, we first identified reciprocal BLAST best hits between *L. flavolineata* and *Drosophila melanogaster* proteins using BLAST+ v.2.12.0 (Camacho et al., 2009). 8266/11,258 (73.4%) *L. flavolineata* genes possessed a reciprocal best hit with *D. melanogaster*. GO annotations for each *D. melanogaster* gene were acquired from BioMart (Smedley et al., 2009), and each *L. flavolineata* gene was assigned the GO terms of its reciprocal best hit. GO enrichment analysis was then performed in R via the topGO package (Alexa & Rahnenführer, 2009) using TopGO's weight01 algorithm and Fisher's exact test to identify GO terms that were significantly overrepresented (*p* < .01) in a focal set of genes against a background consisting of all genes that appeared in the relevant analysis. Numbers of GO terms reported in the results include only Biological Process terms, but Tables S1–S16 include significant terms from all three ontologies (Biological Process, Cellular Component and Molecular Function).

### 2.4 Gene co‐expression network analysis

We sought to identify co‐expressed modules of genes associated with correlates of risky foraging activity among non‐reproductives. To achieve this, weighted gene co‐expression network analysis was performed in R using the WGCNA package (Langfelder & Horvath, 2008). As WGCNA is particularly sensitive to genes with low expression, data were first subjected to a second round of filtering in which genes that had <10 reads in >90% of sampled individuals were removed, as recommended by the package authors. This second round of filtering removed an additional 1821 genes, leaving a total of 9428 genes. Counts were subjected to DESeq2's variance‐stabilizing transformation prior to further analysis. Consensus gene modules across all non‐reproductives were then constructed using a soft‐threshold power of 6 and the signed hybrid adjacency criterion. Network summary measures and gene dendrograms for this analysis are provided in Figures S1 and S2. Initially, 26 gene modules were identified. Modules whose eigengene correlation was >75% were subsequently merged, after which 20 consensus modules remained. Finally, the Pearson correlation of each module eigengene with each phenotypic trait was calculated and subjected to Benjamini–Hochberg FDR correction.

### 2.5 Comparison with an independent evolutionary origin of sociality

As this is the first transcriptomic dataset generated for any stenogastrine wasp species, we wished to assess the degree to which the genes associated with reproductive division of labour in this species were the same as those associated with sociality in a separate lineage. There is no facultatively social vespine or polistine wasp (Taylor et al., 2018); thus, we compared our stenogastrine wasp data with that of the closest independently evolved social insect species for which data were available, the European paper wasp *Polistes dominula* (Taylor et al., 2021) which has become a model organism for social organization in the Vespidae. We hypothesised that, if there is a common evolutionary pathway to evolving reproductive division of labour, we might expect to see some degree of overlap in transcriptomic signals between these two independent lineages, even though they differ in their form of social commitment (colony life being facultative in *L. flavolineata* and obligate in *P. dominula*). Conversely, there may be little congruence in the molecular processes underpinning similar phenotypes in these two lineages due to their independent origins and/or their different forms of sociality. To test this hypothesis, we obtained orthologues between *L. flavolineata* and *P. dominula*. BLASTing our new *L. flavolineata* genome against the Pdom_r1.2 reference genome (Standage et al., 2016) using BLAST+ v.2.12.0 (Camacho et al., 2009), we defined orthologues as reciprocal best BLAST hits between the two species. We then used Fisher's exact tests in R to quantify the overlap between sets of DEGs identified in this study and the set of genes identified as differentially expressed between reproductives and helpers in *P. dominula* using DESeq2 by Taylor et al. (2021).

## 3 RESULTS

### 3.1 *L. flavolineata* genome assembly

Illumina sequencing generated 163 million pairs of genomic PE reads and 102 million pairs of MP reads. The GC content distribution was uniform, indicating that there was no contamination in the sequenced tissue. The size of the genome estimated based on 17‐mers was 373 Mbp. The assembled genome sequence obtained with SOAPdenovo was 291 Mbp, counting only contigs larger than 500 bp. The longest scaffold in the assembly had a length of 5.23 Mbp, and the N50 scaffold length was 1.5 Mbp (Table S1). Of 4415 BUSCO groups searched, 97.9% were found in the assembly (96.9% complete). We therefore conclude that this assembly is a comprehensive representation of the *L. flavolineata* genome in terms of regions encoding gene sequences.

### 3.2 Phenotypic correlates of rank in *L. flavolineata*

**FIGURE 1:**
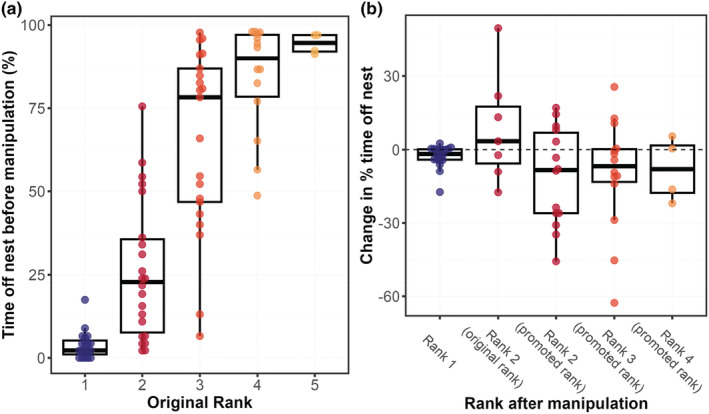
(a) Proportion of time spent off‐nest before manipulation (*n* = 83 individuals) and (b) change in proportion of time spent off‐nest following manipulation (*n* = 61 individuals) by individuals of different within‐colony ranks.

We first verified variation in role‐related phenotypes along the reproductive hierarchy by measuring ovarian development and time spent off‐nest of individual wasps with different reproductive ranks. In unmanipulated colonies, there was a strongly significant negative relationship between within‐colony age rank and the amount of time individuals from unmanipulated colonies spent off the nest (linear regression of arcsine square root‐transformed proportional time off‐nest on rank: slope ± SE = 0.34 ± 0.024, *p* < .001, *R* ^2^ = .724; Figure 1a; Table S2). Intriguingly, there was high variance in time spent off‐nest among individuals in Ranks 2–4, with Rank 3 individuals in particular exhibiting a nearly bimodal distribution in times spent off nest. While we did not sequence DNA and so cannot confirm kinship between individuals, it is possible that individuals that spent more time helping at a given rank did so because they were more closely related than others to the Rank 1 individual (for example, sisters vs. cousins; Bridge & Field, 2007).

We also observed the expected changes in foraging efforts in response to manipulation of rank. Individuals that were promoted in rank by removing a higher‐ranked individual reduced their time off the nest (and therefore putatively the time spent foraging) following manipulation (*n* = 35; two‐sided Wilcoxon test *W* = 137, *p* = .017), although the dispersion of this change was high (mean ± SD of per‐individual change in time off nest = −10.16 ± 20.25%; Figure 1b). By contrast, individuals whose rank was not manipulated showed no significant shift in their time spent off the nest (*n* = 26; mean ± SD = 0.35 ± 12.68; two‐sided Wilcoxon *W* = 90, *p* = .2; Figure 1b), despite the fact that colony size was decreased for these individuals following manipulation. This indicates that the removal of brood during manipulation was successful in maintaining a constant per‐capita foraging requirement and supports the interpretation that behavioural changes in individuals with manipulated ranks were indeed due to their shift in the reproductive hierarchy.

Ovarian development was strongly dependent on rank at time of removal. The most dominant individual (Rank 1) within a nest was always inseminated and possessed several mature eggs in her ovarioles (*n* = 19; mean ± SD = 12.63 ± 2.29 eggs). By contrast, individuals of Rank 2 and below (*n* = 64) almost never possessed developed eggs at time of removal and were never found to be inseminated (Table S2).

Overall, these results are in line with previous work (Bridge & Field, 2007; Field et al., 1999, 2006; Shreeves & Field, 2002; Sumner et al., 2002), showing that *L. flavolineata* colonies are defined by a binary reproductive division of labour between individuals of Rank 1 (who are the sole egg layers) and Rank 2 and beyond (non‐reproductives). An age‐based hierarchy exists among the non‐reproductives, with reduced investment in foraging effort for higher‐ranked individuals in the queue. We therefore posit that time spent off the nest can be taken as a proxy for time spent foraging and hereafter refer to the proportion of time spent off the nest by each individual as that individual's ‘foraging effort’.

### 3.3 Reproductive division of labour predicts gene expression variation

**FIGURE 2:**
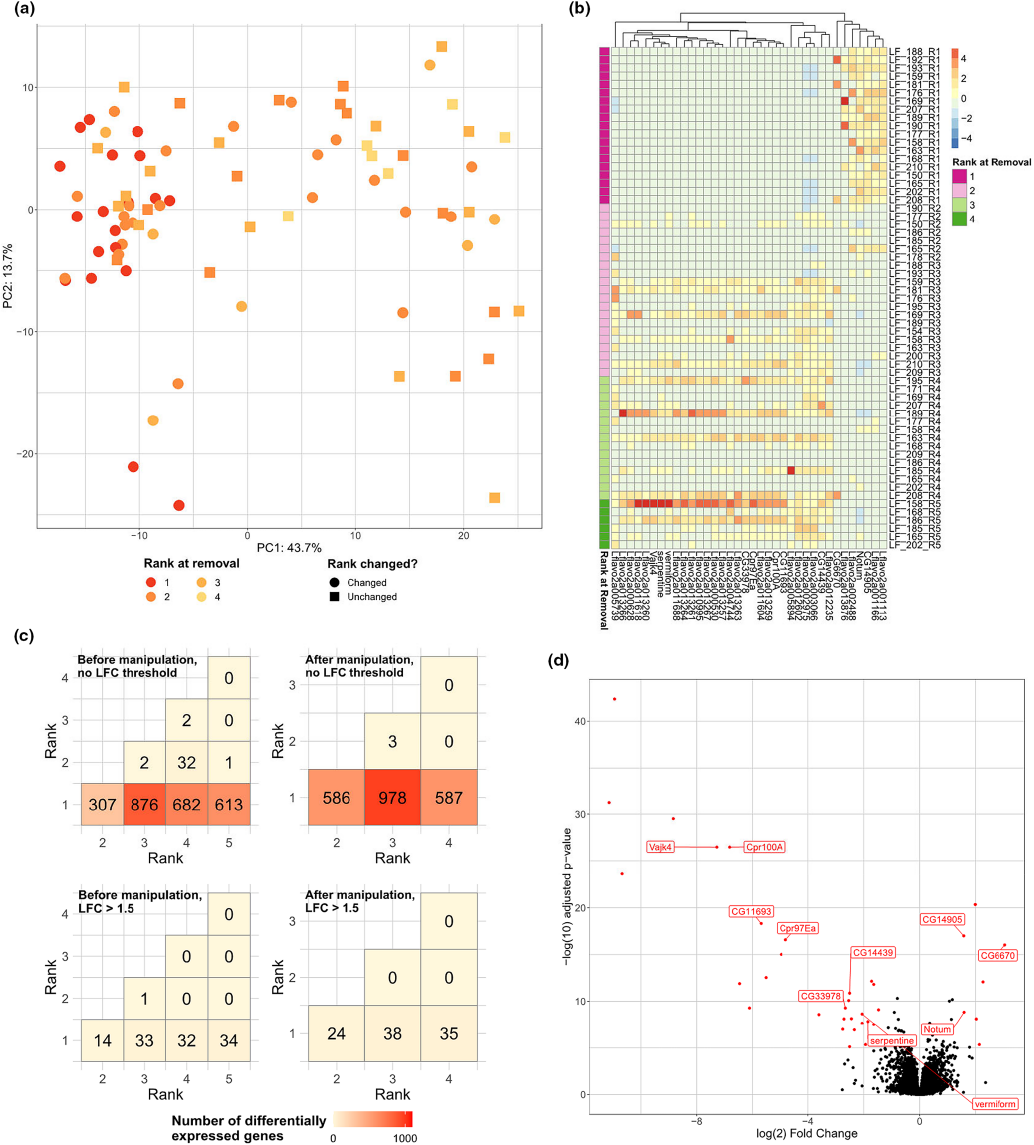
(a) PCA of logged expression values of the 1000 most variably expressed genes among all individuals (*n* = 81 individuals). (b) Heatmap of scaled expression for 36 genes differentially expressed between reproductives and non‐reproductives against a baseline fold change of 1.5. Cell colours represent centred and scaled per‐individual expression levels (red = upregulation; blue = downregulation). Where available, gene IDs given on x‐axis are of the gene's reciprocal best hit in *D. melanogaster*. (c) Number of genes differentially expressed between each pair of ranks. Ranks shown are those to which individuals belonged before (left) and after (right) manipulation. Top two plots: no fold change threshold; Bottom two plots: at a fold change threshold of 1.5. Post‐manipulation rank sample sizes: R1 = 19; R2 = 21; R3 = 15; R4 = 6. Pre‐manipulation rank sample sizes: R1 = 19; R2 = 22; R3 = 21; R4 = 15; R5 = 6. (d) Volcano plot depicting expression of genes in reproductives vs. all post‐manipulation non‐reproductives. Differentially expressed genes (DEGs) are in red. DEGs with a reciprocal best BLAST hit in *D. melanogaster* are annotated with the ID of that hit.

We next sequenced RNA from a number of focal individuals to identify patterns of gene expression associated with within‐colony rank. RNA was successfully sequenced from brain tissue taken from 83 individuals from 28 different nests, with an average of 14.4 M successfully mapped reads per sample. A PCA of all samples using the 1000 most highly variable genes indicated that, while reproductive individuals clustered closely on PC1 (which explained 43.7% of variance) and rank broadly decreased moving along this PC, individuals of non‐reproductive ranks were much more dispersed and many overlapped with the space occupied by reproductives (Figure 2a). These results therefore indicate that the degree of differentiation between reproductives and non‐reproductives in this species may be more subtle than that present in other social lineages, such as *Polistes* (Taylor et al., 2021).

Comparing all reproductives (Rank 1; *n* = 19) against all non‐reproductives (Ranks 2–5; *n* = 64) with DESeq2, we identified 1117 differentially expressed genes (DEGs). The 483 genes that were upregulated in reproductives (Rank 1) were associated with 9 significantly enriched GO terms (Tables S3 and S4), including several terms related to DNA replication and histone binding. The 664 genes that were upregulated in non‐reproductives (Ranks 2–5) were associated with 30 significantly enriched GO terms (Tables S5 and S6), most of which related to respiration and metabolism. However, repeating this analysis with a log(2) fold change threshold of 1.5 yielded a much smaller set of 36 DEGs. Moreover, a heatmap of these 36 genes' expression levels showed that expression of each gene was variable both within and between ranks, and that for some genes Rank 2 individuals exhibited expression closer to the mean for Rank 1 than for lower ranks (Figure 2b). These results are therefore consistent with the possibility that reproductive differentiation in *Liostenogaster* is less complete than that observed in *Polistes*. Eleven of these 36 DEGs between reproductives and non‐reproductives possessed a reciprocal BLAST best hit in *D. melanogaster*, including a number of genes involved in chitin binding (*serpentine*, *vermiform*, *Vajk4*, and *Cpr100A*) all of which were upregulated in non‐reproductives, and two genes involved in germline stem cell differentiation (*CG14905* and *CG6670*), both upregulated in reproductives.

We found significant signals of differential expression between Rank 1 individuals and individuals of each of the other ranks, with >300 DEGs in each comparison (Figure 2c), but as above the number of DEGs decreased by an order of magnitude when applying a fold change threshold. Patterns of differential expression held whether grouping individuals by their rank prior to manipulation or by their rank following manipulation (Figure 2c), indicating that the degree of differentiation between reproductives and non‐reproductives was not significantly changed by manipulation.

### 3.4 Effects of social hierarchy on brain transcription

#### 3.4.1 Absolute foraging effort explains gene expression variation among non‐reproductives better than rank

We next focused on patterns of gene expression associated with the phenotypic variation observed within nests' hierarchies. Over 1000 genes were identified as being differentially expressed with foraging effort and/or with rank, regardless of whether the rank considered was that identified before or after manipulation (Table 1). However, much of this differential expression was driven by reproductive individuals, which exhibit the highest rank and lowest foraging rates (Figure 1a). Excluding these individuals from the analysis and thereby focusing exclusively on non‐reproductives, the number of DEGs identified with rank and/or foraging effort was 1–3 orders of magnitude lower (Table 1).

**TABLE 1:** Number of differentially expressed genes (DEGs) whose expression was correlated with continuous traits across all individuals or when excluding Rank 1 individuals.

Trait	DEGs (rank 1 included)	DEGs (rank 1 excluded)
Original rank	1204	45
Manipulated rank	1085	1
Foraging effort	1282	256
Age	NA	0

*Note*: Age data were not available for Rank 1 individuals, and so it was not possible to identify genes differentially expressed with age when including this category; however, no effect of age was detected for the set of wasps of known ages. Pre‐manipulation *n* = 64 non‐reproductive and 19 reproductive wasps; post‐manipulation *n* = 42 non‐reproductive and 19 reproductive wasps.

Analysis of these non‐reproductive individuals (*n* = 64 wasps) revealed that foraging effort explains differences in gene expression more strongly than rank: 256 genes were correlated with foraging effort but only 45 genes with rank (Table 1). Furthermore, no genes were found to be differentially expressed with the residuals of rank on foraging effort, while 18 genes were differentially expressed with the residuals of foraging effort on rank, which suggests that it is foraging rate per se rather than rank that best predicts individual gene expression profiles. Eight of these 18 genes possessed a reciprocal best hit in *D. melanogaster*, including three genes (*CG11693*, *Cpr97Ea* and *Vajk4*) that were positively correlated with foraging effort and also very strongly upregulated in non‐reproductives compared to reproductives (Figure 2d). Both *Cpr97Ea* and *Vajk4* are involved in chitin‐based cuticle development. Also included among these eight orthologues, and positively correlated with foraging effort, were an odorant binding protein gene, *Obp83*, and a gene involved in motor neuron axon development, *sidestep*.

There was no detectable effect of age itself on gene expression among non‐reproductive individuals (Table 1), despite a substantial age range of 20 days between the oldest and youngest individuals in this analysis. This is a somewhat surprising result given that age is known to have a strong effect on gene expression patterns in other species (e.g. Lockett et al., 2016; Lucas et al., 2017), including among non‐reproductives in *P. dominula* (Taylor et al., 2021). Because of this finding, subsequent analyses focused on the molecular signatures of foraging effort among non‐reproductive individuals.

We next explored the functions of the genes (*n* = 256) that were putatively associated with foraging. Among non‐reproductives, 173 genes were upregulated with respect to foraging rate. These were associated with 23 significantly enriched GO terms, many of which related to developmental, metabolic and respiratory processes (Tables S7 and S8). The 83 genes that were downregulated with respect to foraging rate were associated with 9 GO terms, including several terms related to metabolism or visual perception (Tables S9 and S10).

Individuals that were promoted in rank after experimental removal of a higher‐ranked wasp responded by modulating their foraging effort to match their new rank, and this change appears to have been matched at the level of gene expression. Individuals that were promoted from Rank 3 to Rank 2 exhibited a significant degree of gene expression differentiation when compared to Rank 3 individuals that had not changed their rank: 218 genes separated these two groups, and this set of DEGs overlapped significantly with those identified as being differentially expressed with foraging rate among non‐reproductives (Fisher's exact test *p* = .016; Table S11). Meanwhile, individuals that were promoted to Rank 2 showed little differentiation from Rank 2 individuals that had not changed their rank (10 DEGs; Table S12). These results suggest that the shifts in investment in individual‐level foraging effort that accompany a change in rank are also reflected at the level of the brain transcriptome, with individuals adopting a transcriptional profile that is closer to their new rank than to the rank that they possessed prior to promotion.

#### 3.4.2 Distinct suites of co‐expressed genes are associated with foraging effort

**FIGURE 3:**
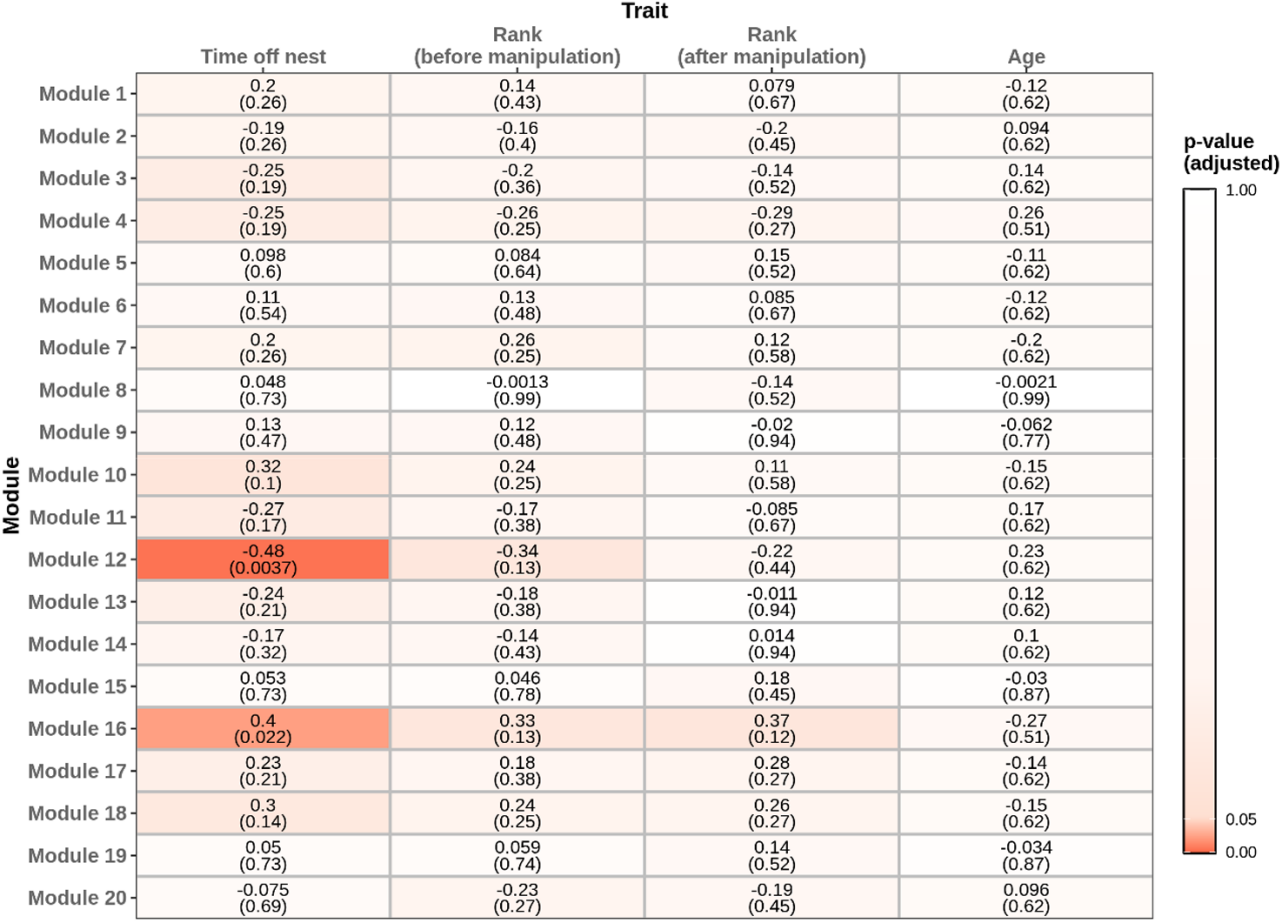
Association of phenotypic traits (time off nest, which is a proxy for foraging effort; rank assessed before manipulation; rank assessed after manipulation; and age) among non‐reproductive individuals with co‐expressed gene modules present in those individuals. Number outside parentheses: Pearson correlation. Number within parentheses: FDR‐corrected *p*‐value.

Across the brain transcriptomes of all non‐reproductive individuals (*n* = 64), we identified 20 distinct co‐regulated gene modules ranging in size from 32 to 1831 genes. None of these modules showed expression levels correlated with rank (either before or after manipulation) or with age, but two exhibited strong (but opposing) correlations with foraging effort (Figure 3).

One module (Module 12; *n* = 83 genes) was significantly negatively associated with foraging rate (*r* = −.48, *p* = .004). Genes that were more strongly correlated with foraging rate, whether positively or negatively, were also more strongly correlated with the eigengene of Module 12 (i.e. they had higher ‘module membership’), suggesting a meaningful relationship between the expression of this module and individuals' foraging effort (Figure S3). Furthermore, genes that were part of this module overlapped significantly both with genes that were found to be negatively correlated with respect to foraging rate when using DESeq2 (two‐sided hypergeometric test *p* < .001), and also with genes that were reproductive‐biased (i.e. those that were upregulated in reproductives vs. non‐reproductives; Fisher's exact test FDR‐adjusted *p* = .016). However, we did not find this same pattern of overlap at the level of putative gene function. The 83 genes contained within Module 12 were enriched for 13 GO terms, several of them associated with visual processing (Tables S13 and S14), but these terms did not overlap significantly with those that were reproductive‐biased (Fisher's exact test FDR‐adjusted *p* = 1) or with those that were negatively correlated with foraging rate (Fisher's exact test FDR‐adjusted *p* = .116).

A second, larger module (Module 16; *n* = 606 genes) exhibited a strongly positive association with foraging rate (*r* = .4, *p* = .022). Genes that were more strongly associated with foraging effort were also more strongly correlated with this module's eigengene (Figure S3). The genes in this module were associated with 43 GO terms, including many terms associated with respiration, and metabolic and biosynthetic processes (Tables S15 and S16). Contrary to Module 12, both genes and GO terms associated with Module 16 overlapped significantly with the set of genes and terms that were upregulated with foraging rate among non‐reproductives, as measured by DESeq2 (genes: Fisher's exact test FDR‐adjusted *p* < .001; GO terms: Fisher's exact test FDR‐adjusted *p* < .001), and genes and GO terms that were upregulated in non‐reproductives versus reproductives (genes: Fisher's exact test FDR‐adjusted *p* < .001; GO terms: Fisher's exact test FDR‐adjusted *p* < .001).

### 3.5 Comparison with reproductive DEGs in *P. dominula*

In order to determine whether sets of genes associated with reproductive roles in *P. dominula* overlapped significantly with genes associated with reproduction and/or helping activity in *L. flavolineata*, we obtained a total of 8460 reciprocal best BLAST hits between *L. flavolineata* and *P. dominula* and then compared our results to those obtained for the comparison between reproductives and non‐reproductives in Taylor et al. (2021).

**FIGURE 4:**
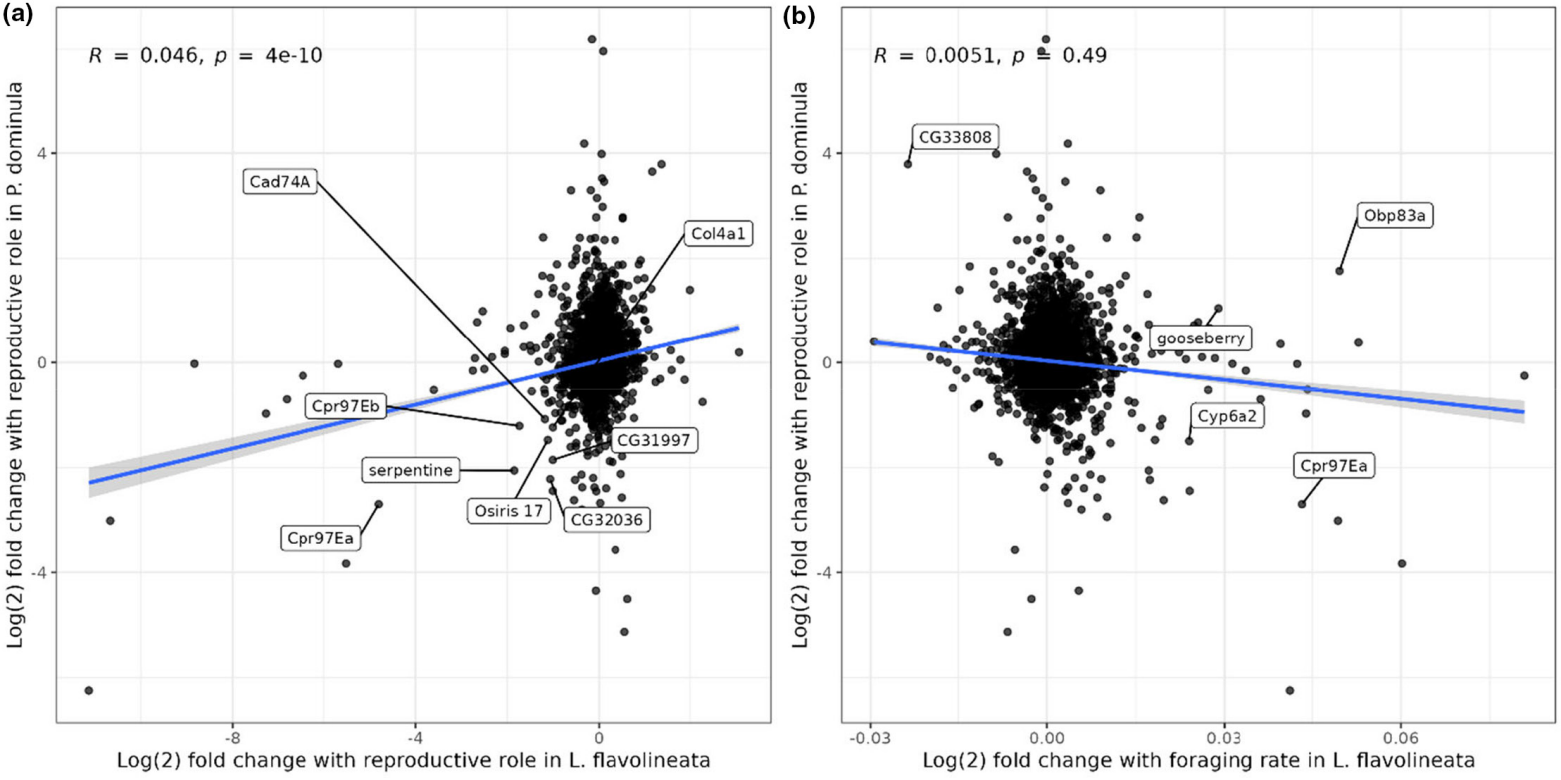
(a) DESeq2 gene expression fold changes between *L. flavolineata* reproductives and non‐reproductives (*x*‐axis) and *P. dominula* reproductives and non‐reproductives (*y*‐axis). Genes with a reciprocal best hit in *D. melanogaster* and absolute fold change >1.5 in both species annotated. (b) DESeq2 gene expression fold changes with increasing foraging effort among *L. flavolineata* non‐reproductives (*x*‐axis) and *P. dominula* reproductives and non‐reproductives (*y*‐axis). Genes with a reciprocal best hit in *D. melanogaster*, absolute fold change >1.5 in *P. dominula*, and >0.02 in *L. flavolineata* annotated. Blue lines: fit based on linear model. *N* = 8460 genes with a reciprocal BLAST best hit between the two species. *P. dominula* data taken from Taylor et al. (2021).

The fold changes of gene expression between reproductives and non‐reproductives were weakly but significantly correlated between these species (*R* = .046; Kendall rank correlation *p* < .001; Figure 4a). By contrast, when performing correlation tests with 100 random permutations of the same fold changes as a null model, the mean correlation was close to 0 (Kendall's tau = .0013; *p* = .49), indicating that the correlation between species was not obtained by chance alone. Intriguingly, this overlap between species appears to be primarily driven by non‐reproductives: while there was a significant overlap in the set of genes upregulated in non‐reproductives in the two species (101 genes overlapping from lists of lengths 542 and 824; Fisher's exact test FDR‐adjusted *p* < .0001), the same was not true of the set of genes upregulated in reproductives in the species (57 genes overlapping from lists of lengths 469 and 1038; Fisher's exact test FDR‐adjusted *p* = .34). We identified eight genes that were strongly upregulated (fold change > log2(1.5)) in non‐reproductives of both species while also possessing a reciprocal best hit in *D. melanogaster*. These again included a number of genes associated with chitin binding: *serpentine*, *Cpr97Eb* and *Cpr97Ea* (Figure 4a).

We also performed the same set of comparisons using the set of genes we identified as differentially expressed with foraging effort among non‐reproductives in *L. flavolineata*. Fold changes for these comparisons were not correlated overall (Kendall's tau = .0051; *p* = .49; Figure 4b), but again we found strongly divergent signals for genes upregulated in different directions: genes whose expression was positively correlated with foraging effort in *L. flavolineata* were significantly enriched among non‐reproductive‐biased genes in *P. dominula* (288 genes overlapping from lists of lengths 542 and 488; Fisher's exact test FDR‐adjusted *p* = .005), but negatively foraging‐associated genes did not exhibit a significant overlap with those that were reproductive‐biased in *P. dominula* (10 genes overlapping from lists of lengths 469 and 83; Fisher's exact test FDR‐adjusted *p* = .68). We identified five genes with reciprocal *D. melanogaster* best BLAST hits that displayed particularly large absolute log(2) fold changes in both species (>1.5 between *P. dominula* reproductive roles and >0.02 with *L. flavolineata* foraging effort; note that because foraging effort is on a numeric scale, fold changes with this variable are necessarily much smaller than for a binary comparison). Three of these genes showed concordant expression in the two species: one, *CG33808*, is a nucleosome component upregulated in *P. dominula* reproductives and negatively correlated with foraging rate in *L. flavolineata*, while the other two (*Cyp6a2* and *Cpr97Ea*) were upregulated in *P. dominula* non‐reproductives and positively associated with foraging rate in *L. flavolineata* (Figure 4b). The remaining two genes were not only upregulated in *P. dominula* reproductives but also positively correlated with foraging rate in *L. flavolineata*: *gooseberry* is involved in synaptic activity, and *Obp83a* is an odorant binding protein.

## 4 DISCUSSION

Caste differences in obligately social insect species are developmentally stable and associated with strong signatures of molecular differentiation (e.g. He et al., 2019; Morandin et al., 2015; Qiu et al., 2022; Warner et al., 2019). Whether such patterns of molecular differentiation are also present between reproductives and non‐reproductives in species that exhibit a high degree of social plasticity is less clear, especially outside the few facultatively social bee species that have been studied, with tentative evidence against this hypothesis in *Polistes* (Qiu et al., 2018). Our results indicate that patterns of molecular differentiation in the social colonies of *L. flavolineata*, a facultatively social wasp that represents an independent and little‐studied origin of sociality, resemble those found in the colonies of obligately social insects, though with a greater degree of variability among individuals sharing the same reproductive role. Of the three genes that were strongly upregulated in reproductives and possessed reciprocal best BLAST hits in *D. melanogaster*, two have previously been identified as being involved in germline stem cell differentiation in flies (Tan et al., 2018). This is a surprising finding given that we sequenced brains rather than reproductive tissue but might indicate that these genes play a role in cellular redifferentiation in a multitude of tissues rather than solely in gonads.

In this species, as in other social insect taxa, the strongest signatures of within‐colony differentiation are found between reproductives and non‐reproductives, despite the presence of significant behavioural variation among non‐reproductives. Given a prima facie expectation that brain transcriptomes should track behavioural states, it may seem surprising that we were able to detect such significant differences, because the behaviour of Rank 2 individuals in this species is intermediate between that of reproductives (Rank 1 individuals) which do not forage at all (Cant & Field, 2001; Field & Foster, 1999; Shreeves & Field, 2002) and low‐ranked non‐reproductives which perform the majority of the colony's foraging (Figure 1a). However, this finding is in agreement with a number of studies that have indicated a strong relationship between ovarian activation and brain gene expression (Amdam et al., 2006; Wang et al., 2009, 2010), and reinforces the notion that ovarian activation is the defining characteristic of division of labour in social insects.

Focusing on transcriptomic differences among non‐reproductives, we identified approximately 250 genes whose expression varied in line with foraging rate—a substantial number, though significantly smaller than that associated with reproductive role differences for the same fold change threshold. A plausible null hypothesis is that this expression variation simply reflects phenotypic variation among individuals; for example, differential expression with foraging rate might stem from the energetic demands of spending more time in flight. However, we believe that three factors speak against this null hypothesis and are in favour of the alternative hypothesis that gene expression variation among non‐reproductives reflects variation in investment into helping at the nest.

First, if within‐colony gene expression differences primarily reflect differences in energetic expenditure, then we should have observed greater differences between Rank 2 individuals and Rank 4/Rank 5 individuals than between Rank 2 individuals and Rank 1 reproductives, who are more similar in foraging effort (Figure 1a), yet this was not the case (Figure 2). Second, individuals that were promoted from Rank 3 to Rank 2 shifted gene expression to match their new rank, which suggests that gene expression variation is not primarily structured by independent factors such as age. Third, the majority (17/25) of the GO terms that were enriched among foraging‐biased genes were also significantly upregulated in non‐reproductives over reproductives. Most of these shared GO terms related to metabolic processes such as GO:0009063:*cellular amino acid catabolic process* and GO:0009055:*electron transfer activity*, a pattern similar to that observed in role‐biased GO terms in the paper wasp *Polistes dominula* (Standage et al., 2016; Taylor et al., 2021). This seems to belie the possibility that these genes were solely associated with such specific behaviours as flying and foraging. In fact, GO terms for visual processing were enriched among those genes that were negatively associated with foraging rate, a surprising result given that foragers presumably rely on visual signals to locate prey. Negatively foraging‐associated genes associated with visual processing included *trpl* and *Arr2*, both of which are strongly associated with photoreceptor activity in flies. Fine‐scale facial markings play a role in communicating reproductive status in *L. flavolineata* (Baracchi et al., 2013), and we therefore speculate that higher‐ranked individuals upregulate genes associated with photoreception in order to better navigate the upper social echelons of the colony by assessing these subtle visual cues. Although we acknowledge that GO annotations are frequently patchy and incomplete, overall these factors are consistent with the hypothesis that gene expression variation among *L. flavolineata* non‐reproductives genuinely represents variation in individuals' potential direct fitness (i.e. their likelihood of achieving a reproductive position in the near future), over and above simply reflecting differences in age or energy expenditure.

Although we hypothesise that it is prospective reproductive opportunities rather than energetic expenditure alone that explains the variation in gene expression observed among non‐reproductives, we identified a larger number of genes differentially expressed with foraging rate than with rank. Moreover, the set of rank‐biased genes was almost entirely subsumed by the set of foraging‐biased genes. This may indicate that rank is in fact a relatively low‐resolution indicator of individual‐level variation in proximity to the reproductive role. Within‐rank variation in the foraging rate of Rank 2 and Rank 3 individuals was very high relative to that of Rank 1 individuals (Figure 1a), which suggests that factors other than rank influence individuals' foraging effort. Nest size is known to be one such factor (Cant & English, 2006; Shreeves & Field, 2002), but others might include the age, health and fecundity of the incumbent reproductive (Bridge & Field, 2007; Kokko & Johnstone, 1999), or relatedness between a given helper and the incumbent (e.g. a sibling vs. a cousin). If individuals modulate their foraging rate based on the likelihood of future inheritance of the reproductive position, and that likelihood is affected by the projected mortality of the current reproductive, then observed foraging rate might actually be a stronger proxy of individual‐level reproduction versus helping than rank itself.

We found small but significant overlaps in the genes underlying reproductive division of labour in *L. flavolineata* and in *P. dominula*, a species representing an independent origin of wasp sociality and a separate mode of social living (one in which social nesting is facultative and another in which social nesting is obligate). Genes that were non‐reproductive‐biased in *P. dominula* were also more likely than expected by chance to be positively correlated with foraging effort in *L. flavolineata*, although it should be noted that the number of overlapping genes was low and represented only a small proportion of the total differential expression present in each species. It is particularly interesting that we observed stronger overlaps between the two species when considering non‐reproductive biased genes as opposed to reproductive‐biased genes. This is opposite to the pattern that we might have expected given results from species with developmentally committed reproductive castes, in which queen‐biased genes are more strongly conserved than worker‐biased genes (Harpur et al., 2014; Qiu et al., 2022; Warner et al., 2019). That we found apparently greater conservation of non‐reproductive‐biased gene expression profiles in the two taxa we studied may indicate that the opposite is true for species such as *P. dominula* and *L. flavolineata* that lack committed castes and therefore retain conflict over reproductive role differentiation within colonies. This result therefore reinforces the fact that results generated using species with developmentally committed castes such as *Apis* honey bees and ants should not be assumed to be applicable to social insect species that lack such castes (Boomsma, 2022; Boomsma & Gawne, 2018). Moreover, while we found significant overlaps between the two species on which we focused, these overlaps were very small in absolute terms. This is consistent with there being a great deal of flexibility between lineages in terms of the molecular mechanisms by which reproductive division of labour can evolve (Rehan & Toth, 2015; Sumner et al., 2023; Wyatt et al., 2023).

Genes implicated in chitin binding and cuticle development appeared in all groups of genes associated with non‐reproductives. One gene, in particular, *Cuticular protein 97Ea* (*Cpr97Ea*), arose repeatedly, being strongly upregulated in non‐reproductives over reproductives in both *L. flavolineata* and *P. dominula*, and also positively associated with foraging rate among *L. flavolineata* non‐reproductives. In *Drosophila*, this gene is associated with larval cuticle development, which makes it somewhat surprising that we found it to be consistently associated with fitness role differences in adults in two separate wasp lineages. While we cannot rule out the possibility of contamination from cuticular tissue during dissections, such an explanation is unlikely given that the degree of contamination would not be expected to correlate with social roles. Elias‐Neto et al. (2014) and Falcon et al. (2019) have raised the possibility that ongoing cuticle development in adult social insects might be dependent on social context rather than age alone, and our finding that associated genes were upregulated with foraging rate and reproductive role (but not with age alone) appears to support this possibility in the two social wasp lineages that we studied. However, our analyses were based on brain tissue, which raises the question of whether these nominally cuticular genes might play a role in other functions, such as cognition. In fact, all of the chitin‐associated genes that we found to be upregulated in non‐reproductives (*Cpr97Ea*, *serpentine*, *vermiform*, *Vajk4* and *Cpr100A*) appear to be active within the same uncharacterised cell cluster of the *Drosophila* brain atlas (Davie et al., 2018). While we currently lack data to confirm whether an equivalent cell cluster exists in the *L. flavolineata* brain, this combination of results suggests that these genes may have a behavioural function that goes beyond their role in chitin binding.

Facultatively social insects are important models with which to investigate the earliest stages of insect social evolution, in which individuals can switch opportunistically between direct and indirect fitness strategies. Colonies in the facultatively social hover wasp *L. flavolineata* are characterized by linear hierarchies, and an individual's position in the resulting ‘queue’ dictates her investment into altruistic foraging behaviour, a system that lends itself well to studies of the molecular correlates of alternative fitness strategies. In this study, we have shown that gene expression in *L. flavolineata* colonies is structured by both actualised and potential direct fitness, that individuals are able to facultatively shift their gene expression profiles to match changes in direct fitness prospects, and that distinct co‐expression modules are associated with reproductive versus helping fitness strategies. These results represent the first in‐depth study of the molecular basis of social behaviour in a vespid species outside of the Polistinae, the only such analysis for a facultatively social wasp, and the first genome sequence for a Stenogastrine wasp, representing an independent evolutionary lineage of insect sociality. These resources and the insights provided by our analyses open fruitful lines for future research into the origin of condition‐dependent altruism, and reveal co‐expressed signals of transcriptomic differentiation that appear to track differential investment into reproductive versus non‐reproductive roles.

## AUTHOR CONTRIBUTIONS

DT and SS conceived of and designed the experiments. DT carried out the experiments and performed dissections and RNA extractions. AB‐S, FCF, NS‐P, HH and RG performed genome assembly and annotation. BAT conducted the analyses with advice from MR and SS. BAT wrote the original manuscript and designed the figures. All authors read and contributed to the manuscript.

## CONFLICT OF INTEREST STATEMENT

All authors declare that they have no conflicts of interest.

## BENEFIT‐SHARING STATEMENT

Our data and results have been made available in public databases as described in the previous section. Our research is compliant with all relevant local laws. Sample collections were conducted under research and collecting permits from the Malaysian Economic Planning Unit.

## Supporting information


Figures S1–S3.

Tables S1–S16.

Appendix S1.

Appendix S2.

Appendix S3.

## Data Availability

Genome assembly files are archived under BioProject ID PRJNA542636. Transcriptomic sequence data with associated sample metadata are archived under BioProject ID PRJNA801104. Scripts used in the analysis can be accessed at https://github.com/Sumner‐lab/LF2022.

## References

[mec17217-bib-0001] Alexa, A. , & Rahnenführer, J. (2009). Gene set enrichment analysis with topGO. Bioconductor Improv, 27, 1–26.

[mec17217-bib-0002] Amdam, G. V. , Csondes, A. , Fondrk, M. K. , & Page, R. E. (2006). Complex social behaviour derived from maternal reproductive traits. Nature, 439(7072), 76–78. 16397498 10.1038/nature04340 PMC2665028

[mec17217-bib-0003] Andrews, S. (2010). FastQC: A quality control tool for high throughput sequence data [Online] . http://www.bioinformatics.babraham.ac.uk/projects/fastqc/

[mec17217-bib-0004] Bank, S. , Sann, M. , Mayer, C. , Meusemann, K. , Donath, A. , Podsiadlowski, L. , Kozlov, A. , Petersen, M. , Krogmann, L. , Meier, R. , Rosa, P. , Schmitt, T. , Wurdack, M. , Liu, S. , Zhou, X. , Misof, B. , Peters, R. S. , & Niehuis, O. (2017). Transcriptome and target DNA enrichment sequence data provide new insights into the phylogeny of vespid wasps (Hymenoptera: Aculeata: Vespidae). Molecular Phylogenetics and Evolution, 116, 213–226. 28887149 10.1016/j.ympev.2017.08.020

[mec17217-bib-0005] Baracchi, D. , Petrocelli, I. , Cusseau, G. , Pizzocaro, L. , Teseo, S. , & Turillazzi, S. (2013). Facial markings in the hover wasps: Quality signals and familiar recognition cues in two species of Stenogastrinae. Animal Behaviour, 85(1), 203–212. 10.1016/j.anbehav.2012.10.027

[mec17217-bib-0006] Bolger, A. M. , Lohse, M. , & Usadel, B. (2014). Trimmomatic: a flexible trimmer for Illumina sequence data. Bioinformatics, 30(15), 2114–2120. 24695404 10.1093/bioinformatics/btu170 PMC4103590

[mec17217-bib-0007] Boomsma, J. J. (2022). Domains and major transitions of social evolution. Oxford University Press.

[mec17217-bib-0008] Boomsma, J. J. , & Gawne, R. (2018). Superorganismality and caste differentiation as points of no return: How the major evolutionary transitions were lost in translation. Biological Reviews, 93(1), 28–54. 28508537 10.1111/brv.12330

[mec17217-bib-0009] Bridge, C. , & Field, J. (2007). Queuing for dominance: Gerontocracy and queue‐jumping in the hover wasp *Liostenogaster flavolineata* . Behavioral Ecology and Sociobiology, 61(8), 1253–1259.

[mec17217-bib-0010] Camacho, C. , Coulouris, G. , Avagyan, V. , Ma, N. , Papadopoulos, J. , Bealer, K. , & Madden, T. L. (2009). BLAST+: Architecture and applications. BMC Bioinformatics, 10(1), 421. 10.1186/1471-2105-10-421 20003500 PMC2803857

[mec17217-bib-0011] Cant, M. A. , & English, S. (2006). Stable group size in cooperative breeders: The role of inheritance and reproductive skew. Behavioral Ecology, 17(4), 560–568.

[mec17217-bib-0012] Cant, M. A. , & Field, J. (2001). Helping effort and future fitness in cooperative animal societies. Proceedings of the Royal Society of London, Series B: Biological Sciences, 268(1479), 1959–1964. 10.1098/rspb.2001.1754 PMC1088835 11564355

[mec17217-bib-0013] Davie, K. , Janssens, J. , Koldere, D. , De Waegeneer, M. , Pech, U. , Kreft, Ł. , Aibar, S. , Makhzami, S. , Christiaens, V. , Bravo González‐Blas, C. , Poovathingal, S. , Hulselmans, G. , Spanier, K. I. , Moerman, T. , Vanspauwen, B. , Geurs, S. , Voet, T. , Lammertyn, J. , Thienpont, B. , … Aerts, S. (2018). A single‐cell transcriptome atlas of the aging Drosophila brain. Cell, 174(4), 982–998. 29909982 10.1016/j.cell.2018.05.057 PMC6086935

[mec17217-bib-0014] Dobin, A. , Davis, C. A. , Schlesinger, F. , Drenkow, J. , Zaleski, C. , Jha, S. , Batut, P. , Chaisson, M. , & Gingeras, T. R. (2013). STAR: ultrafast universal RNA‐seq aligner. Bioinformatics, 29(1), 15–21. 23104886 10.1093/bioinformatics/bts635 PMC3530905

[mec17217-bib-0015] Dohm, J. C. , Minoche, A. E. , Holtgräwe, D. , Capella‐Gutiérrez, S. , Zakrzewski, F. , Tafer, H. , Rupp, O. , Sörensen, T. R. , Stracke, R. , Reinhardt, R. , Goesmann, A. , Kraft, T. , Schulz, B. , Stadler, P. F. , Schmidt, T. , Gabaldón, T. , Lehrach, H. , Weisshaar, B. , & Himmelbauer, H. (2014). The genome of the recently domesticated crop plant sugar beet (*Beta vulgaris*). Nature, 505(7484), 546–549. 10.1038/nature12817 24352233

[mec17217-bib-0016] Elias‐Neto, M. , Nascimento, A. L. , Bonetti, A. M. , Nascimento, F. S. , Mateus, S. , Garófalo, C. A. , & Bitondi, M. M. (2014). Heterochrony of cuticular differentiation in eusocial corbiculate bees. Apidologie, 45, 397–408.

[mec17217-bib-0017] Ewels, P. A. , Peltzer, A. , Fillinger, S. , Patel, H. , Alneberg, J. , Wilm, A. , Garcia, M. U. , Di Tommaso, P. , & Nahnsen, S. (2020). The nf‐core framework for community‐curated bioinformatics pipelines. Nature Biotechnology, 38(3), 276–278. 10.1038/s41587-020-0439-x 32055031

[mec17217-bib-0069] Falcon, T. , Pinheiro, D. G. , Ferreira‐Caliman, M. J. , Turatti, I. C. C. , Abreu, F. C. P. , Galaschi‐Teixeira, J. S. , Martins, J. R. , Elias‐Neto, M. , Soares, M. P. M. , Laure, M. B. , Figueiredo, V. L. C. , Lopes, N. P. , Simões, Z. L. P. , Garófalo, C. A. , & Bitondi, M. M. G. (2019). Exploring integument transcriptomes, cuticle ultrastructure, and cuticular hydrocarbons profiles in eusocial and solitary bee species displaying heterochronic adult cuticle maturation. PloS One, 14(3), e0213796. 10.1371/journal.pone.0213796 30870522 PMC6417726

[mec17217-bib-0018] Field, J. (2008). The ecology and evolution of helping in hover wasps (Hymenoptera: Stenogastrinae). In J. Korb & J. Heinze (Eds.), Ecology of social evolution (pp. 85–107). Springer Berlin Heidelberg.

[mec17217-bib-0019] Field, J. , Cronin, A. , & Bridge, C. (2006). Future fitness and helping in social queues. Nature, 441(7090), 214–217. 16688175 10.1038/nature04560

[mec17217-bib-0020] Field, J. , & Foster, W. (1999). Helping behaviour in facultatively eusocial hover wasps: An experimental test of the subfertility hypothesis. Animal Behaviour, 57(3), 633–636. 10196053 10.1006/anbe.1999.0995

[mec17217-bib-0021] Field, J. , Shreeves, G. , & Sumner, S. (1999). Group size, queuing and helping decisions in facultatively eusocial hover wasps. Behavioral Ecology and Sociobiology, 45(5), 378–385.

[mec17217-bib-0022] Field, J. , Shreeves, G. , Sumner, S. , & Casiraghi, M. (2000). Insurance‐based advantage to helpers in a tropical hover wasp. Nature, 404(6780), 869–871. 10786792 10.1038/35009097

[mec17217-bib-0023] Foster, K. R. , Wenseleers, T. , & Ratnieks, F. L. (2006). Kin selection is the key to altruism. Trends in Ecology & Evolution, 21(2), 57–60. 16701471 10.1016/j.tree.2005.11.020

[mec17217-bib-0024] Gurevich, A. , Saveliev, V. , Vyahhi, N. , & Tesler, G. (2013). QUAST: Quality assessment tool for genome assemblies. Bioinformatics, 29(8), 1072–1075. 23422339 10.1093/bioinformatics/btt086 PMC3624806

[mec17217-bib-0025] Hamilton, W. D. (1963). The evolution of altruistic behavior. The American Naturalist, 97(896), 354–356.

[mec17217-bib-0026] Harpur, B. A. , Kent, C. F. , Molodtsova, D. , Lebon, J. M. , Alqarni, A. S. , Owayss, A. A. , & Zayed, A. (2014). Population genomics of the honey bee reveals strong signatures of positive selection on worker traits. Proceedings of the National Academy of Sciences of the United States of America, 111(7), 2614–2619. 24488971 10.1073/pnas.1315506111 PMC3932857

[mec17217-bib-0027] He, X. J. , Jiang, W. J. , Zhou, M. , Barron, A. B. , & Zeng, Z. J. (2019). A comparison of honeybee (*Apis mellifera*) queen, worker and drone larvae by RNA‐seq. Insect Science, 26(3), 499–509. 29110379 10.1111/1744-7917.12557

[mec17217-bib-0028] Jones, B. M. , Kingwell, C. J. , Wcislo, W. T. , & Robinson, G. E. (2017). Caste‐biased gene expression in a facultatively eusocial bee suggests a role for genetic accommodation in the evolution of eusociality. Proceedings of the Royal Society B: Biological Sciences, 284(1846), 20162228. 10.1098/rspb.2016.2228 PMC5247497 28053060

[mec17217-bib-0029] Kapheim, K. M. , Jones, B. M. , Pan, H. , Li, C. , Harpur, B. A. , Kent, C. F. , Zayed, A. , Ioannidis, P. , Waterhouse, R. M. , Kingwell, C. , Stolle, E. , Avalos, A. , Zhang, G. , McMillan, W. O. , & Wcislo, W. T. (2020). Developmental plasticity shapes social traits and selection in a facultatively eusocial bee. National Academy of Sciences of the United States of America, 117, 13615–13625. 10.1073/pnas.2000344117 PMC7306772 32471944

[mec17217-bib-0030] Kapheim, K. M. , Smith, A. R. , Ihle, K. E. , Amdam, G. V. , Nonacs, P. , & Wcislo, W. T. (2012). Physiological variation as a mechanism for developmental caste‐biasing in a facultatively eusocial sweat bee. Proceedings of the Royal Society B: Biological Sciences, 279(1732), 1437–1446. 10.1098/rspb.2011.1652 PMC3282364 22048951

[mec17217-bib-0031] Kokko, H. , & Johnstone, R. A. (1999). Social queuing in animal societies: A dynamic model of reproductive skew. Proceedings of the Royal Society of London, Series B: Biological Sciences, 266(1419), 571–578.

[mec17217-bib-0032] Kopylova, E. , Noé, L. , & Touzet, H. (2012). SortMeRNA: Fast and accurate filtering of ribosomal RNAs in metatranscriptomic data. Bioinformatics, 28(24), 3211–3217. 10.1093/bioinformatics/bts611 23071270

[mec17217-bib-0033] Kovaka, S. , Zimin, A. V. , Pertea, G. M. , Razaghi, R. , Salzberg, S. L. , & Pertea, M. (2019). Transcriptome assembly from long‐read RNA‐seq alignments with StringTie2. Genome Biology, 20(1), 1–13. 31842956 10.1186/s13059-019-1910-1 PMC6912988

[mec17217-bib-0034] Kronauer, D. J. , & Libbrecht, R. (2018). Back to the roots: The importance of using simple insect societies to understand the molecular basis of complex social life. Current Opinion in Insect Science, 28, 33–39. 30551765 10.1016/j.cois.2018.03.009

[mec17217-bib-0035] Krueger, F. (2015). Trim galore: A wrapper tool around Cutadapt and FastQC to consistently apply quality and adapter trimming to FastQ files . https://github.com/FelixKrueger/TrimGalore

[mec17217-bib-0036] Langfelder, P. , & Horvath, S. (2008). WGCNA: An R package for weighted correlation network analysis. BMC Bioinformatics, 9(1), 559. 19114008 10.1186/1471-2105-9-559 PMC2631488

[mec17217-bib-0037] Langmead, B. , & Salzberg, S. L. (2012). Fast gapped‐read alignment with Bowtie 2. Nature Methods, 9(4), 357–359. 22388286 10.1038/nmeth.1923 PMC3322381

[mec17217-bib-0038] Leadbeater, E. , Carruthers, J. M. , Green, J. P. , Rosser, N. S. , & Field, J. (2011). Nest inheritance is the missing source of direct fitness in a primitively eusocial insect. Science, 333(6044), 874–876. 21836014 10.1126/science.1205140

[mec17217-bib-0039] Lockett, G. A. , Almond, E. J. , Huggins, T. J. , Parker, J. D. , & Bourke, A. F. (2016). Gene expression differences in relation to age and social environment in queen and worker bumble bees. Experimental Gerontology, 77, 52–61. 26883339 10.1016/j.exger.2016.02.007

[mec17217-bib-0040] Love, M. I. , Huber, W. , & Anders, S. (2014). Moderated estimation of fold change and dispersion for RNA‐seq data with DESeq2. Genome Biology, 15(12), 550. 25516281 10.1186/s13059-014-0550-8 PMC4302049

[mec17217-bib-0041] Lucas, E. R. , Romiguier, J. , & Keller, L. (2017). Gene expression is more strongly influenced by age than caste in the ant Lasius Niger. Molecular Ecology, 26(19), 5058–5073. 28742933 10.1111/mec.14256

[mec17217-bib-0042] Luo, R. , Liu, B. , Xie, Y. , Li, Z. , Huang, W. , Yuan, J. , He, G. , Chen, Y. , Pan, Q. , Liu, Y. , Tang, J. , Wu, G. , Zhang, H. , Shi, Y. , Liu, Y. , Yu, C. , Wang, B. , Lu, Y. , Han, C. , … Wang, J. (2012). SOAPdenovo2: An empirically improved memory‐efficient short‐read de novo assembler. GigaScience, 1(1), 18. 23587118 10.1186/2047-217X-1-18 PMC3626529

[mec17217-bib-0043] Marçais, G. , & Kingsford, C. (2011). A fast, lock‐free approach for efficient parallel counting of occurrences of k‐mers. Bioinformatics, 27(6), 764–770. 21217122 10.1093/bioinformatics/btr011 PMC3051319

[mec17217-bib-0044] Morandin, C. , Brendel, V. P. , Sundström, L. , Helanterä, H. , & Mikheyev, A. S. (2019). Changes in gene DNA methylation and expression networks accompany caste specialization and age‐related physiological changes in a social insect. Molecular Ecology, 28(8), 1975–1993. 30809873 10.1111/mec.15062

[mec17217-bib-0045] Morandin, C. , Dhaygude, K. , Paviala, J. , Trontti, K. , Wheat, C. , & Helanterä, H. (2015). Caste‐biases in gene expression are specific to developmental stage in the ant *Formica exsecta* . Journal of Evolutionary Biology, 28(9), 1705–1718. 26172873 10.1111/jeb.12691

[mec17217-bib-0046] Patalano, S. , Vlasova, A. , Wyatt, C. , Ewels, P. , Camara, F. , Ferreira, P. G. , Asher, C. L. , Jurkowski, T. P. , Segonds‐Pichon, A. , Bachman, M. , González‐Navarrete, I. , Minoche, A. E. , Krueger, F. , Lowy, E. , Marcet‐Houben, M. , Rodriguez‐Ales, J. L. , Nascimento, F. S. , Balasubramanian, S. , Gabaldon, T. , … Sumner, S. (2015). Molecular signatures of plastic phenotypes in two eusocial insect species with simple societies. Proceedings of the National Academy of Sciences of the United States of America, 112(45), 13970–13975. 26483466 10.1073/pnas.1515937112 PMC4653166

[mec17217-bib-0047] Patro, R. , Duggal, G. , Love, M. I. , Irizarry, R. A. , & Kingsford, C. (2017). Salmon provides fast and bias‐aware quantification of transcript expression. Nature Methods, 14(4), 417–419. 28263959 10.1038/nmeth.4197 PMC5600148

[mec17217-bib-0048] Qiu, B. , Dai, X. , Li, P. , Larsen, R. S. , Li, R. , Price, A. L. , Ding, G. , Texada, M. J. , Zhang, X. , Zuo, D. , Gao, Q. , Jiang, W. , Wen, T. , Pontieri, L. , Guo, C. , Rewitz, K. , Li, Q. , Liu, W. , Boomsma, J. J. , & Zhang, G. (2022). Canalized gene expression during development mediates caste differentiation in ants. Nature Ecology & Evolution, 6(11), 1753–1765. 36192540 10.1038/s41559-022-01884-y PMC9630140

[mec17217-bib-0049] Qiu, B. , Larsen, R. S. , Chang, N. C. , Wang, J. , Boomsma, J. J. , & Zhang, G. (2018). Towards reconstructing the ancestral brain gene‐network regulating caste differentiation in ants. Nature Ecology & Evolution, 2(11), 1782–1791. 30349091 10.1038/s41559-018-0689-x PMC6217981

[mec17217-bib-0050] Rehan, S. M. , & Toth, A. L. (2015). Climbing the social ladder: The molecular evolution of sociality. Trends in Ecology & Evolution, 30(7), 426–433. 26051561 10.1016/j.tree.2015.05.004

[mec17217-bib-0051] Shell, W. A. , & Rehan, S. M. (2018). Behavioral and genetic mechanisms of social evolution: Insights from incipiently and facultatively social bees. Apidologie, 49(1), 13–30.

[mec17217-bib-0052] Shreeves, G. , & Field, J. (2002). Group size and direct fitness in social queues. The American Naturalist, 159(1), 81–95. 10.1086/324125 18707402

[mec17217-bib-0053] Simão, F. A. , Waterhouse, R. M. , Ioannidis, P. , Kriventseva, E. V. , & Zdobnov, E. M. (2015). BUSCO: Assessing genome assembly and annotation completeness with single‐copy orthologs. Bioinformatics, 31(19), 3210–3212. 26059717 10.1093/bioinformatics/btv351

[mec17217-bib-0054] Smedley, D. , Haider, S. , Ballester, B. , Holland, R. , London, D. , Thorisson, G. , & Kasprzyk, A. (2009). BioMart–biological queries made easy. BMC Genomics, 10(1), 22. 19144180 10.1186/1471-2164-10-22 PMC2649164

[mec17217-bib-0055] Smith, A. R. , Kapheim, K. M. , O'Donnell, S. , & Wcislo, W. T. (2009). Social competition but not subfertility leads to a division of labour in the facultatively social sweat bee *Megalopta genalis* (Hymenoptera: Halictidae). Animal Behaviour, 78(5), 1043–1050.

[mec17217-bib-0056] Standage, D. S. , Berens, A. J. , Glastad, K. M. , Severin, A. J. , Brendel, V. P. , & Toth, A. L. (2016). Genome, transcriptome and methylome sequencing of a primitively eusocial wasp reveal a greatly reduced DNA methylation system in a social insect. Molecular Ecology, 25(8), 1769–1784. 26859767 10.1111/mec.13578

[mec17217-bib-0057] Sumner, S. , Casiraghi, M. , Foster, W. , & Field, J. (2002). High reproductive skew in tropical hover wasps. Proceedings of the Royal Society of London, Series B: Biological Sciences, 269(1487), 179–186. 10.1098/rspb.2001.1884 PMC1690882 11798434

[mec17217-bib-0058] Sumner, S. , Favreau, E. , Geist, K. , Toth, A. L. , & Rehan, S. M. (2023). Molecular patterns and processes in evolving sociality: Lessons from insects. Philosophical Transactions of the Royal Society B, 378(1874), 20,220,076. 10.1098/rstb.2022.0076 PMC9939270 36802779

[mec17217-bib-0059] Tan, S. W. S. , Yip, G. W. , Suda, T. , & Baeg, G. H. (2018). Small Maf functions in the maintenance of germline stem cells in the *Drosophila* testis. Redox Biology, 15, 125–134. 29245136 10.1016/j.redox.2017.12.002 PMC5730423

[mec17217-bib-0060] Taylor, B. A. , Cini, A. , Wyatt, C. D. , Reuter, M. , & Sumner, S. (2021). The molecular basis of socially mediated phenotypic plasticity in a eusocial paper wasp. Nature Communications, 12(1), 775. 10.1038/s41467-021-21095-6 PMC7859208 33536437

[mec17217-bib-0061] Taylor, D. , Bentley, M. A. , & Sumner, S. (2018). Social wasps as models to study the major evolutionary transition to superorganismality. Current Opinion in Insect Science, 28, 26–32. 30551764 10.1016/j.cois.2018.04.003

[mec17217-bib-0062] Wang, Y. , Amdam, G. V. , Rueppell, O. , Wallrichs, M. A. , Fondrk, M. K. , Kaftanoglu, O. , & Page, R. E., Jr. (2009). PDK1 and HR46 gene homologs tie social behavior to ovary signals. PLoS One, 4(4), e4899. 19340296 10.1371/journal.pone.0004899 PMC2659776

[mec17217-bib-0063] Wang, Y. , Kaftanoglu, O. , Siegel, A. J. , Page, R. E., Jr. , & Amdam, G. V. (2010). Surgically increased ovarian mass in the honey bee confirms link between reproductive physiology and worker behavior. Journal of Insect Physiology, 56(12), 1816–1824. 20688074 10.1016/j.jinsphys.2010.07.013

[mec17217-bib-0064] Warner, M. R. , Qiu, L. , Holmes, M. J. , Mikheyev, A. S. , & Linksvayer, T. A. (2019). Convergent eusocial evolution is based on a shared reproductive groundplan plus lineage‐specific plastic genes. Nature Communications, 10(1), 1–11. 10.1038/s41467-019-10546-w PMC6570765 31201311

[mec17217-bib-0065] Waterhouse, R. M. , Seppey, M. , Simão, F. A. , Manni, M. , Ioannidis, P. , Klioutchnikov, G. , Kriventseva, E. V. , & Zdobnov, E. M. (2018). BUSCO applications from quality assessments to gene prediction and phylogenomics. Molecular Biology and Evolution, 35(3), 543–548. 29220515 10.1093/molbev/msx319 PMC5850278

[mec17217-bib-0066] West‐Eberhard, M. J. (1975). The evolution of social behavior by kin selection. The Quarterly Review of Biology, 50(1), 1–33.

[mec17217-bib-0067] Wilson, E. O. (1990). Success and dominance in ecosystems: The case of the social insects (Vol. 2, pp. I–XXI). Ecology Institute.

[mec17217-bib-0068] Wyatt, C. D. R. , Bentley, M. A. , Taylor, D. , Favreau, E. , Brock, R. E. , Taylor, B. A. , Favreau, E. , Brock, R. E. , Taylor, B. A. , Bell, E. , Leadbeater, E. , & Sumner, S. (2023). Social complexity, life‐history and lineage influence the molecular basis of castes in vespid wasps. Nature Communications, 14(1), 1046. 10.1038/s41467-023-36456-6 PMC9958023 36828829

