# Supplementary material for: Molecular signatures of alternative reproductive strategies in a facultatively social hover wasp
Source: Mol Ecol. 2023 Nov 28;33(2):e17217. doi: 10.1111/mec.17217 (PMC10953455; doi:10.1111/mec.17217)

Figure S1. WGCNA summary network measures against soft thresholding power. Numbers in the plots indicate the corresponding soft thresholding powers.

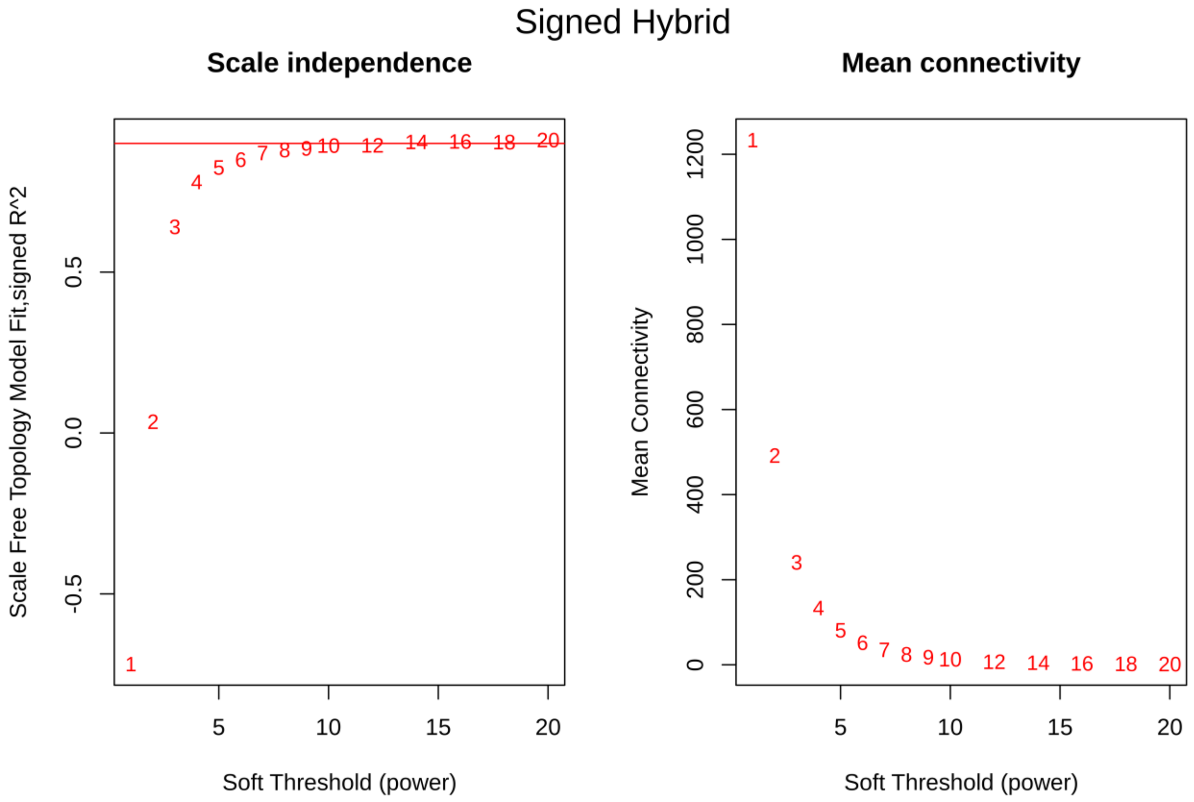

Figure S2. Gene dendrogram with clustering based on consensus topological overlap. Upper colour row: consensus module assignments prior to merging of modules with similar expression profiles. Lower colour row: consensus modules following merging of similar modules.

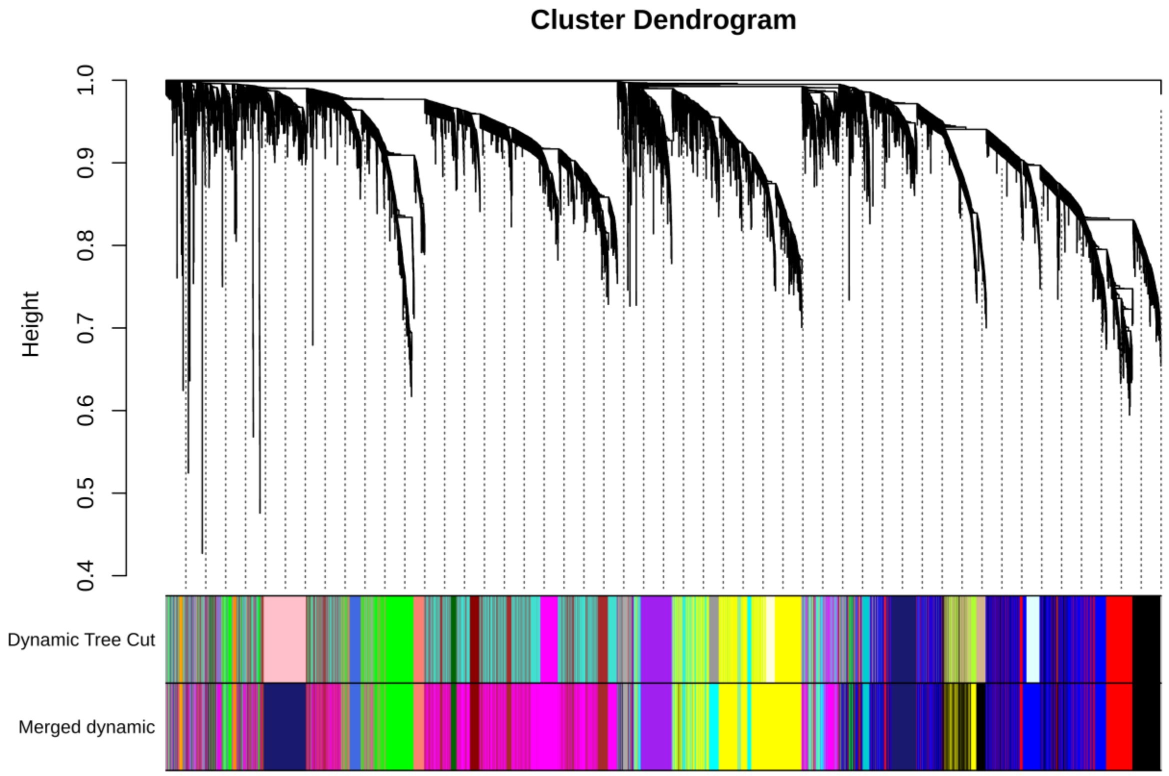

Figure S3. Strength of absolute (unsigned) correlation of genes with foraging effort among non-reproductives, plotted against module membership of those genes within (a) Module 12 (n = 83 genes) and (b) Module 16 (n = 606 genes).

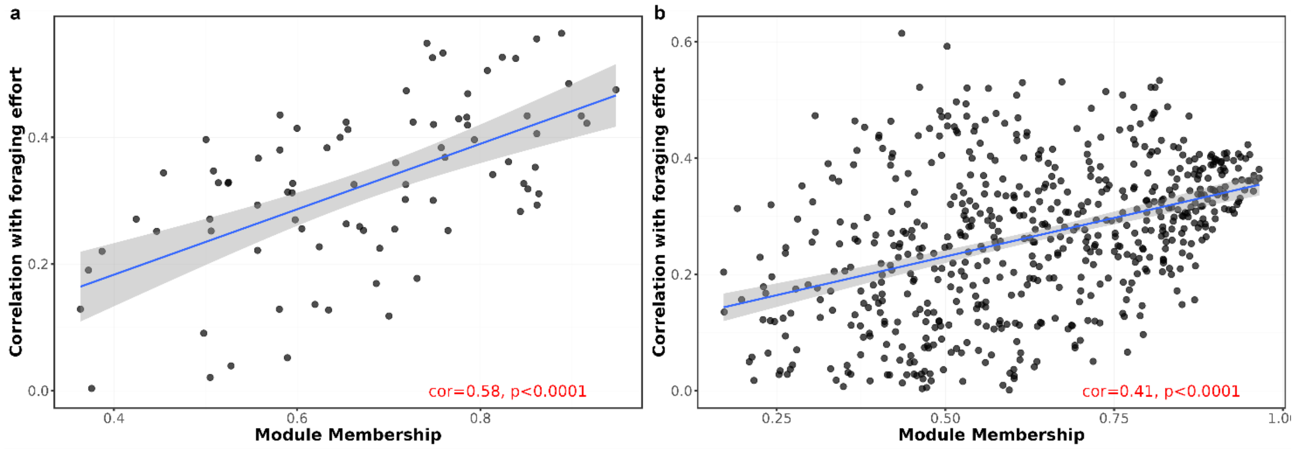

Supplement: Supplementary file 1 — Figures S1–S3. [file MEC-33-0-s005.pdf]
